# Global segregation of cortical activity and metastable dynamics

**DOI:** 10.3389/fnsys.2015.00119

**Published:** 2015-08-25

**Authors:** Peter Stratton, Janet Wiles

**Affiliations:** ^1^Queensland Brain Institute, The University of QueenslandBrisbane, QLD, Australia; ^2^Centre for Clinical Research, The University of QueenslandBrisbane, QLD, Australia; ^3^School of Information Technology and Electrical Engineering, The University of QueenslandBrisbane, QLD, Australia

**Keywords:** thalamocortical matrix, autonomous metastable dynamics, cortical segregation, default mode network, sleep, wakefulness, complexity, spiking networks

## Abstract

Cortical activity exhibits persistent metastable dynamics. Assemblies of neurons transiently couple (integrate) and decouple (segregate) at multiple spatiotemporal scales; both integration and segregation are required to support metastability. Integration of distant brain regions can be achieved through long range excitatory projections, but the mechanism supporting long range segregation is not clear. We argue that the thalamocortical matrix connections, which project diffusely from the thalamus to the cortex and have long been thought to support cortical gain control, play an equally-important role in cortical segregation. We present a computational model of the diffuse thalamocortical loop, called the competitive cross-coupling (CXC) spiking network. Simulations of the model show how different levels of tonic input from the brainstem to the thalamus could control dynamical complexity in the cortex, directing transitions between sleep, wakefulness and high attention or vigilance. The model also explains how mutually-exclusive activity could arise across large portions of the cortex, such as between the default-mode and task-positive networks. It is robust to noise but does not require noise to autonomously generate metastability. We conclude that the long range segregation observed in brain activity and required for global metastable dynamics could be provided by the thalamocortical matrix, and is strongly modulated by brainstem input to the thalamus.

## Introduction

In wakefulness and in rapid eye movement (REM) sleep, cortical activity exhibits persistent ongoing complex dynamics (Breakspear et al., 2003; Honey et al., 2007). In this state, activity shifts continuously throughout the cortex, and cortical regions couple (integrate) and decouple (segregate) across multiple spatial and temporal scales (Sporns et al., 2000a; Varela et al., 2001; Shanahan, 2008; Tognoli and Kelso, 2014). Each individual episode of integration and segregation is transient, but a continuous superposition of such episodes through space and time results in the observed persistent metastability of cortical dynamics (Tognoli and Kelso, 2014). This dynamical complexity is hypothesized to support the brain's flexibility and sophisticated processing capabilities (Breakspear et al., 2003; Buzsáki and Draguhn, 2004; Fries, 2005; Tognoli and Kelso, 2014), including memory retrieval, planning and problem solving (Binder et al., 1999; Mazoyer et al., 2001). During times when the brain is not actively processing sensory stimuli or task-related events, and as such is in a state known as the “resting” or “default-mode” state, cortical activity is concentrated in a well-defined sub-network including regions of frontal and association cortices (specifically the ventromedial prefrontal cortex, posterior cingulate cortex, ventral precuneus, and parts of the medial temporal and medial, lateral and inferior parietal cortices) (Greicius et al., 2003; Uddin et al., 2008). Activity of this default-mode network (DMN) is anticorrelated with activity in much of the rest of the cortex—that is, activation of the DMN and of those cortical centers used for sensory and task-related processing is largely mutually exclusive (Greicius et al., 2003; Uddin et al., 2008; Tomasi and Volkow, 2011).

Metastable cortical states, and the functions these dynamics presumably underpin (Binder et al., 1999; Mazoyer et al., 2001; Breakspear et al., 2003; Buzsáki and Draguhn, 2004; Fries, 2005; Tognoli and Kelso, 2014), cannot exist without the myriad, often overlooked, sub-cortical areas that provide the cortex with controlling and modulatory input. Projections from the pedunculopontine nucleus (PPN) and the laterodorsal tegmental nuclei (LDT), parts of the brainstem network collectively known as the ascending arousal system (AAS—but previously known as the reticular activating system), enter the intralaminar nuclei of the thalamus (IL) and thence on to the cortex through the thalamocortical matrix connections. The AAS is thought to modulate wake and sleep states as well as arousal and vigilance levels (Moruzzi and Magoun, 1949; Reese et al., 1995; Jones, 2003). The IL matrix connections project diffusely and somewhat non-specifically to large portions of the cortex, which in turn project back to the IL through the thalamic reticular nucleus (RN). Notably, these projections have opposing effects: whereas the cortex excites the RN, the RN exerts an inhibitory influence on the IL. Hence, rising global cortical activity increases RN activation, which in turn inhibits the IL, reducing its input to cortical activation and ultimately countering the activity rise in the cortex. Similarly, a decrease in cortical activity can cause an increase in thalamocortical input from the AAS. This diffuse matrix thalamocortical loop can therefore potentially dynamically control overall activity levels in the cortex (Steriade and McCarley, 1990). Effectively, it implements a mechanism similar to *k*-winner-take-all (WTA) across the entire cortex, where the allowed activity level *k* is controlled by AAS input to the IL. WTA networks are known to be able to implement powerful computational functions (Maass, 2000).

**Figure 1:**
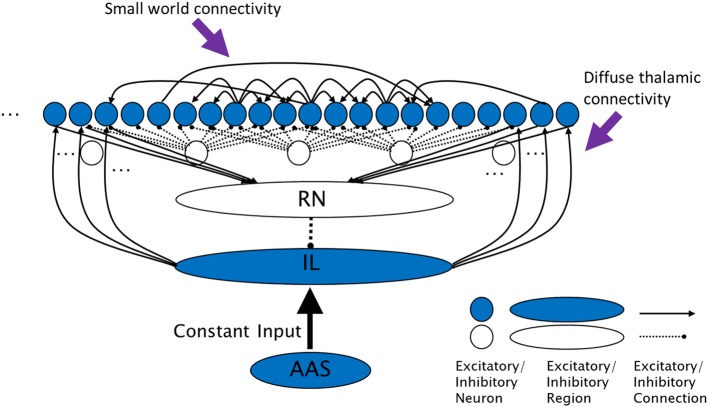
**The complex cross coupling (CXC) spiking network (not all connections shown)**. Filled circles are cortical principal cells (pyramidal neurons). Open circles are inhibitory interneurons. Excitatory cortical pyramidal neurons are innervated by diffuse thalamic (intralaminar nuclei) projections (from shaded oval). The intralaminar nuclei receive continuous steady, not time-varying, input current from the AAS. Cortical neurons project back to the thalamic reticular nucleus which inhibits the intralaminar nuclei, completing the diffuse thalamocortical loop. Cortical neurons excite each other within a small neighborhood radius, inhibit each other through local interneurons within a larger inhibitory radius, and project a small number of random long range excitatory synapses to other cortical neurons. RN, thalamic reticular nucleus; IL, diffuse (intralaminar) thalamic nuclei; AAS, ascending arousal system.

Brain integration is served by long range excitatory connections, but the paucity of long range inhibition in the brain has meant that the mechanisms to support long range segregation of brain activity are less obvious and are not well understood. Long range inhibition has been discovered within the visual cortex (McDonald and Burkhalter, 1993), between the hippocampus and entorhinal cortex (Melzer et al., 2012), and between the prefrontal cortex and nucleus accumbens (Lee et al., 2014). However, currently these long range inhibitory connections are known to exist only in or between a limited number of specific structures; they certainly do not approach the abundance of the long range excitatory projections forming the large fiber tracts that criss-cross the brain. The mechanism by which, for example, the DMN is segregated from other cortical regions is unclear (Greicius et al., 2003; Uddin et al., 2008). The contribution of the AAS, IL and RN to global cortical activation control is widely suspected (Steriade and McCarley, 1990; Taylor and Farrukh, 1996; Saper et al., 2001). In this paper we refine this view and argue for the specific functions of long range competition and segregation of cortical activity, and thereby the support of cortical metastability. To demonstrate this potential we present a computational model of the diffuse thalamocortical loop, called the Complex Cross Coupling (CXC) spiking network (see Figure 1). The CXC network includes input from the AAS and RN to the IL, local and long range corticocortical connections and local inhibitory interneurons. Simulations demonstrate that the model requires no extrinsic noise to exhibit its full range of dynamical states. We use the model to show how different levels of input from the AAS to the IL could support a range of dynamical states in the cortex, including states with high dynamical complexity.

## Methods

### Neuron and synapse models

All simulations were conducted using the Parallel Circuit Simulator (PCSIM) (Pecevski et al., 2009), a comprehensive software package for the simulation of large neural networks. Simulations used Euler integration with a time-step of 0.1 ms.

The Complex Cross Coupling Spiking Network was constructed using two types of Izhikevich model cortical neurons (regular spiking, RS, and fast spiking, FS) (Izhikevich, 2003). These neurons provide for realistic neuron membrane dynamics such as spike frequency adaptation, intrinsic bursting, resonance and bistability, whilst being computationally tractable for large network simulations (Izhikevich, 2004). The Izhikevich model is defined by three equations over two variables, the membrane potential *v* (nominally in mV) and the membrane recovery variable *q*, which are updated as follows:

(1) $$\begin{array}{rcl} v^{\prime } & = & 0.04v^{2}+5v+140-q+I \end{array}$$

(2) $$\begin{array}{rcl} q^{\prime } & = & a\left(bv-q\right) \end{array}$$

(3) $$\begin{array}{rcl} \text{if}\;v & \geq & 30\;\text{then}\\ \;\;\;\;\;\;v & \leftarrow & g\\ \;\;\;\;\;\;q & \leftarrow & q+h \end{array}$$

where *I* is the summed synaptic input current and *a, b, g*, and *h* are neuron-specific model parameters. In this study, all excitatory neurons were modeled as regular-spiking (RS) cells with the parameters (*a, b, g, h*) = (0.02, 0.2, −65, 8) and inhibitory neurons as fast spiking (FS) cells with (*a, b, g, h*) = (0.1, 0.2, −65, 2). These dimensionless parameters are set as specified in Izhikevich (2003) to obtain membrane dynamics (modeled by *v*) that closely resemble the modeled classes of neuron (i.e., RS and FS cells). In this study, pyramidal cells are modeled as regular spiking, and inhibitory interneurons as fast spiking.

The synapses received presynaptic spikes that would initiate postsynaptic currents that decayed with characteristic time constants. Synapses were conductance-based, meaning they each had a reversal potential *E*_*rev*_ at which current flow ceased and beyond which the direction of current flow reversed. Reversal potential for excitatory and inhibitory synapses was 0 mV and −90 mV respectively.

To implement short term synaptic dynamics (which was applied to synapses between all excitatory neurons in one experiment, the results of which are shown in **Figure 5D**), a combination of synaptic depression and facilitation was used (Markram et al., 1998). For synaptic depression, synaptic efficacy was assumed to be a finite resource, of which a proportion *p* was in use at any given time. Only an amount *u* of the currently-available proportion of synaptic efficacy, (1 – *p*), was used at the occurrence of each spike; *p* recovered back to zero with time constant *d* (*p* was therefore bounded in [0,1)). The rate of change of *p* for synapse *i*, *ṗ*_*i*_, was given by:

(4) $$\begin{array}{l} {ṗ}_{i}=\delta \left(t-t_{i}\right){\left(1-p\right)}_{i}u_{i}-\frac{p_{i}}{d_{i}} \end{array}$$

where δ is the Dirac delta function and *t*_*i*_ was the time of the last spike from the neuron that was presynaptic to synapse *i*. The initial value of *u* for dynamic synapses was set to *U*, then for synaptic facilitation *u* was increased on the occurrence of each spike, recovering back to *U* with time constant *f* (*u* was therefore bounded in [*U*,1)). The rate of change of *u* for synapse *i*, ${\overset{{}^{\circ}}{u}}_{i}$, was given by:

(5) $$\begin{array}{l} {\overset{{}^{\circ}}{u}}_{i}=\delta \left(t-t_{i}\right)\left(1-u_{i}\right)U+\frac{\left(U-u_{i}\right)}{f_{i}} \end{array}$$

The synaptic usage factor *U* was set to 0.15 with a recovery time constant of *f* = 1000 ms for all dynamic synapses. Total synaptic current, *I*_*dynamic*_, for a postsynaptic neuron was given by the sum of all the currents from its afferent synapses:

(6) $$\begin{array}{l} I_{dynamic}=\underset{i}{\sum }\left[w_{i}\left(1-p_{i}\right)u_{i}\left(v-E_{rev}\right)\right] \end{array}$$

where *w*_*i*_ was the total efficacy of synapse *i* and *v* was the postsynaptic membrane potential.

The synaptic recovery time constant *d* was set to 50 ms for local excitatory-to-excitatory and 500 ms for long-range excitatory-to-excitatory connections. The difference was motivated in part by the different recovery times for AMPA and NMDA receptors. Long range connections in this model were loosely associated with feedback connections within the hierarchically-organized, massively recurrent cortical connectome. These feedback connections may be NMDA-rich, as against feedforward and local connections which may be predominantly AMPA-based (Thiele, 2012). The time constants used were longer than typically acknowledged for these receptor types to compensate for the absence of other network influences in the model, such as cholinergic neuromodulation, which are known to enhance NMDAR function.

For static synapses (synapses of fixed efficacy, i.e., with no short term synaptic dynamics), synaptic efficacy was assumed to be an infinite (non-depleting) resource, so *u* was always 1 and the rate of change of *p* for synapse *i*, *ṗ*_*i*_, simplified to:

(7) $$\begin{array}{l} {ṗ}_{i}=\delta \left(t-t_{i}\right)-\frac{p_{i}}{d_{i}} \end{array}$$

Total synaptic current, *I*_*static*_, for a postsynaptic neuron was then:

(8) $$\begin{array}{l} I_{static}=\underset{i}{\sum }\left[w_{i}\left(1-p_{i}\right)\left(v-E_{rev}\right)\right] \end{array}$$

Static synapses were used in all simulations in this study except for **Figure 5D** (for which dynamic synapses were used as above). For static excitatory-to-excitatory synapses the synaptic recovery time constant *d* was set to 50 ms, for excitatory-to-inhibitory 5 ms and for inhibitory-to-excitatory 40 ms. No inhibitory-to-inhibitory connections existed.

### Network

The CXC network was devised for this study in order to investigate the relationship between the diffuse thalamocortical matrix loop and cortical dynamics, although precursor models have been published (Stratton and Wiles, 2010a,b). The CXC model is an abstraction of the essential computational components of the complex thalamocortical connection structure. It utilizes some of the known characteristics of these regions (such as dense local and more sparse long-range connections), while ignoring others (such as cortical layers). As such, it is a general representation only, with no intended specific spatial scale. Its purpose is to show that thalamocortical-like connectivity between simple neuron-like elements can result in non-trivial dynamics, and to point to some of the general neuron and network properties that may be involved.

The network consisted of *n*_*e*_ = 1000 regular-spiking (RS) excitatory pyramidal neurons and *n*_*i*_ = 250 fast-spiking (FS) inhibitory interneurons connected linearly (i.e., in a 1-dimensional network) with directional synapses. Synaptic efficacies were set such that several presynaptic spikes in close succession were required to cause an output spike in a postsynaptic neuron. Connection structure was set to be small world-like, with dense local connectivity and sparse random long range connections, similar to the cortex. Each RS neuron was connected to each of its closest *j* neighbors with local excitatory efficacy *w*_*n*_*/j* where *w*_*n*_ = 2 (the end neurons connected circularly to the opposite end of the neuron vector). Random long-range connections of weight *w*_*r*_*/k* where *w*_*r*_ = *w*_*n*_ were then made from each RS neuron to each other RS neuron with probability *k*/*n*_*e*_, giving an average of *k* long-range connections per neuron. In all simulations *j* = 4 and *k* = 10 (these numbers of connections were smaller than in actual cortex due to the limited size of the modeled network, and synaptic efficacies and currents were therefore scaled up accordingly). Inhibitory FS neurons were spread uniformly between the RS neurons. Each FS neuron received excitatory input from and projected inhibitory output to each of its closest *l* RS neurons with efficacy *w*_*i*_*/l* where *w*_*i*_ = 1 and *l* = 20.

The thalamic reticular (RN) and intralaminar (IL) nuclei were each implemented as a single analog (non-spiking) neuron with output equal to the sum of its input currents. These nuclei are modeled as analog because their function is to balance cortical activity, and while the brain uses spiking neurons in these nuclei, we hypothesize that the summed action of many spikes from many neurons results in this homeostatic balance, so for the CXC model the analog representation is sufficient. RS neurons were innervated by IL projections with synaptic efficacy of 0.4. The IL received continuous steady (not time-varying) input current from the AAS (*I*_*AAS*_) which ranged from 0 to 10 in different experiments. This upper bound on *I*_*AAS*_ was set to strongly depolarize the connected neurons without causing them to reach spiking threshold. Cortical neurons projected to the RN with efficacy 1, with synaptic currents decaying with time constant *d*, as above (Equation 7). The RN inhibited the IL with efficacy −1, completing the diffuse thalamocortical loop. Network activity was initiated by a single input pulse at time zero into a random selection of *n*_*e*_/2 RS neurons. Conduction delays were set in a uniform random distribution between 1 and 25 ms for long range connections and to 1 ms for all other connections.

To test the sensitivity of the results to numerical factors, additional test simulations were conducted with very high temporal resolution (1 μs time-steps) and larger scales (100,000 neurons in the network). Additionally, simulations of 10.000 s duration were run to ensure that network dynamics neither failed nor entered a short limit cycle attractor state. All these simulations exhibited metastable network dynamics across their entire spatiotemporal extents.

### Analysis

Input current from the AAS to the IL (*I*_*AAS*_) was varied from 0 to 10 in steps of 0.01. For each input current level, a simulation was conducted for 10 s of simulated time. Spike times and membrane potentials of all neurons were recorded at each time-step of 0.1 ms. The first 1 s of activity was deemed to be the network settling period during which time the network activity would transition from synchronous bursting (initiated by the input pulse) to sustained activity (or to quiescence depending on the input level). The neural activity for this first second was omitted from further analysis. The remaining 9 s of the recordings were used to calculate the mean firing rate, *r*, of the network (*r* = *T*/*n*_*e*_/9, where *T* is the total number of spikes generated by all RS neurons).

We estimated the local field potential (LFP) that would be recorded from this network at any moment in time, *lfp*(*t*), by summing the membrane potentials of all neurons:

(9) $$\begin{array}{l} lfp\left(t\right)=\underset{m}{\sum }v_{m}\left(t\right) \end{array}$$

where *v*_*m*_(*t*) was the membrane potential of neuron *m* at time *t*. Due to the vertical alignment of cortical dendrites in real brains, real dendritic potentials, when summed from many cortical neurons, contribute to the LFP (Nunez and Srinivasan, 2006). Neurons in this study were simulated as point entities with no dendritic processes, so the neuron (soma) membrane potential was the closest estimate of the dendritic potential. This process of calculating the estimate of the LFP is similar to that used by Beim Graben and Kurths (2008).

An indication of the complexity of the dynamical network state was based on deviations of the interspike interval (ISI) distribution from exponential to multi-modal. During metastable dynamics, three modes (short, medium and long) were evident in the ISI distribution; these modes arose explicitly due to the processes of active integration and segregation in the network. Short ISIs were caused by bursty firing of a neuron, which occurred when the neuron was receiving strong synaptic input from neighboring neurons and from long range connections from distant neurons, and hence indicated that the neuron was being integrated into network activity. Long ISIs were caused by long periods when a neuron did not fire, which occurred when the neuron was receiving weak or no input from neighboring neurons and from long range connections, and hence when the neuron was segregated from network activity.

Spikes firing within the central ISI mode, midway between the short and long modes, were simply following the dominant oscillation frequency of the network, exhibiting neither enhanced integration nor segregation from network activity. Small integration and segregation peaks, when compared to the central peak, therefore indicated that most spikes were entrained to the dominant network oscillation and neuron firing was predominantly periodic, whereas simultaneously-large integration and segregation peaks indicated that the network was in a complex metastable dynamical regime.

Finally, the network *trapping time* was calculated (Marwan et al., 2002). Trapping time quantifies the amount of time a network remains in a given state before transitioning to a new state. The trapping time was determined by first constructing a matrix *S* of states of the network spiking activity over 0.2 s non-overlapping time windows. Within each time window *j*, the number of times each RS neuron *i* emitted a spike was counted:

(10) $$S_{i,j}=\left|\left\{0.2(j-1)\leq t_{i}<0.2j\right\}\right|\text{ for all }i,j$$

where *t*_*i*_ is the set of spike times of neuron *i* and |{…}|denotes the length of a set.

The correlation matrix of *S* was calculated and the resulting matrix was thresholded at 0.5, yielding the state recurrence matrix *R*:

(11) $$\begin{array}{l} R=H\left(\text{corr}\left(S\right)-0.5\right) \end{array}$$

where *H* is the Heaviside step function. *R* is a symmetric matrix that reveals, for all states *j*, which other states were similar (i.e., which other states had a similar pattern of firing neurons). Finally, the mean width of the super-threshold region surrounding the main diagonal of *R* was determined (i.e., for each 0.2 s window represented by an element on the main diagonal of *R*, the width of the super-threshold region around this element was measured perpendicular to the diagonal by stepping outwards from the element until a sub-threshold element, where *H*_*i, j*_ = 0, was found; the mean of these widths for all elements along the main diagonal of *R* is the trapping time of the network). Trapping time is interpreted as follows: If the network transitioned between states rapidly, then adjoining state vectors of *S* had sub-threshold similarity and the diagonal of the recurrence matrix *R* (the trapping time) was only one state wide (i.e., each state was similar only to itself). However, if the state transitions were slow, then adjoining state vectors of *S* had super-threshold similarity and the width around the diagonal of *R* was greater than one. Longer trapping times indicated that the network state was evolving more slowly. Trapping times approaching 10 s (the length of most simulations in this study) indicated that the network dynamics were fixed in an attractor state in which activity did not evolve at all.

## Results

### Autonomous (self-sustained) metastable dynamics

**Figure 2:**
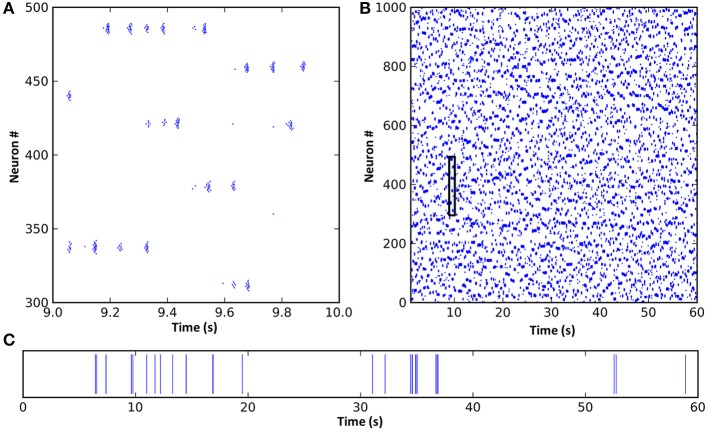
**A spike raster plot of CXC network activity shown at different zoom levels reveals characteristics of complex network dynamics**. These include network-wide oscillations, groups of neighboring neurons unpredictably forming transient oscillating assemblies, and ongoing non-periodic activity. **(A)** Spiking activity of 200 neurons for 1 s. Each horizontal group is an assembly of bursting neurons. Each neuron within an assembly bursts at high frequency, with the bursts occurring every 0.05–0.1 s (10–20 Hz) while the assembly is active. Assemblies are transient, sometimes firing synchronously with other assemblies in the network and sometimes asynchronously. Examples of both synchronous and asynchronous firing can be seen. **(B)** The entire network shown for the full 60 s of simulation. The transient and unpredictable nature of assembly formation and duration is evident. The time period shown in panel **(A)** is boxed. Mean firing rate for all neurons was just 1.0 Hz. **(C)** Spike raster plot for one typical neuron over 60 s, showing random firing with burst activity punctuating extended periods of quiescence. Most neurons had lengthy periods of complete silence lasting 10 s or more despite having mean firing rates around 1 Hz and the network as a whole oscillating at 10–20 Hz.

To establish the baseline complex dynamics supported by the CXC network, constant input from the AAS was first set to the baseline level of 1 and synaptic conductances were set to standard values for the network (see Methods). No noise sources were used within the network or in its input. The network exhibited metastable dynamics (Figure 2) despite being deterministic. We have previously investigated the network properties required for a spiking network (a precursor to the CXC) to exhibit such dynamics (Stratton and Wiles, 2010a,b)—these include small-world or scale-free cortical connectivity, a mechanism of global inhibition, and maintenance of dynamics in a critical (phase transition) state. The parameters of the CXC network of the current study were set to be in the critical region. Metastable network dynamics were robust to moderate network parameter changes, however significant modification of these parameters resulted in either collapse of network dynamics into a limit cycle resembling seizure or failure of activity to propagate causing the total network activity to fall to zero. These two states of seizure and quiescence are the low-complexity states between which the activity in the CXC network rapidly, partially and transiently switched to generate the metastability (Stratton and Wiles, 2010b)^1^. The cortico-thalamo-cortical feedback from the matrix connections played a homeostatic role, lowering cortical input as activity levels increased and raising input as activity levels decreased, ensuring that the network as a whole remained within the critical region of phase space. In all simulations, these autonomous, complex, non-periodic dynamics lasted indefinitely.

Inspection of the firing of neural assemblies (groups of neighboring neurons) in the spike raster plot shows that they occurred approximately 0.05 s apart (Figure 2). This oscillation period was determined by the interplay of the cortico-thalamo-cortical feedback and the time constant of the local synaptic inhibition. Despite the regular oscillation period, these assemblies formed at unpredictable, apparently random times and existed for unpredictable durations (Figure 2; also see Stratton and Wiles, 2010b). Dynamic formation of assemblies was unpredictable because it depended on three tightly coupled factors: (1) which assemblies were currently firing, (2) the precise pattern of random long-range connections from the currently active assemblies, since this pattern would determine which of the currently inactive neurons were receiving strongest synaptic input, and (3) the past history of firing activities, since neurons which had been recently active could still have been in a relative refractory period, meaning other neurons could fire first even if they were receiving weaker synaptic input. Thus, even though the network dynamics were noiseless and deterministic, they were unpredictable unless the entire network state (both static and dynamic, i.e., the entire connectivity map, the complete properties of every neuron and synapse, and the precise current states of all membrane potentials and synaptic currents) was known with absolute accuracy. In other words, the only way to predict the ongoing activity would be to create an exact duplicate of the network, and any inputs impinging upon it, and simulate it in its entirety. (Interestingly, this inability to accurately predict model dynamics for the CXC network applies equally to real brains).

### Integration and segregation

**Figure 3:**
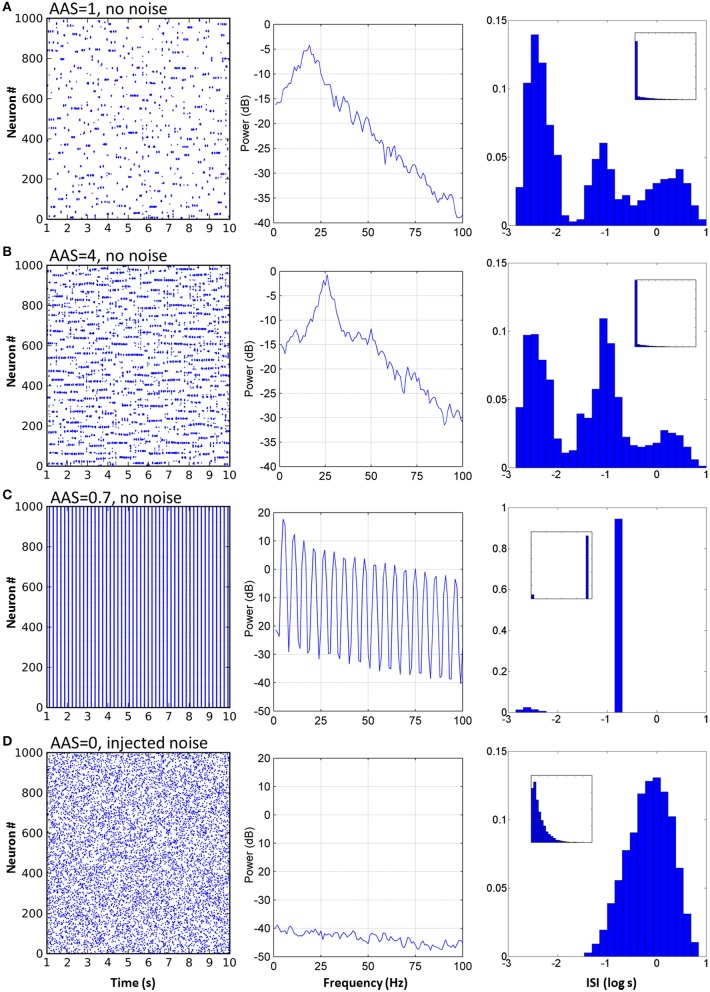
**Varying AAS input induced very different dynamical states in the CXC network**. **(A)** With AAS input set to 1, complex dynamics emerged despite the absence of noise in the system. The spike raster plot for all the principal cells for 10 s of activity is shown (the first 1 s of network settling time was excluded) (left). The Local field potential (LFP) showed a dominant 19 Hz oscillation (center). Firing rate variability was high; neural activity occurred in combinations of strong bursts and long periods of inactivity. The ISI distribution, shown as a probability density, was therefore multi-modal (right), indicative of integration (bursts), and segregation (extended periods of inactivity—see text). Displaying ISI with log time enhances resolution for short ISIs (main panel, right; inset on right shows the ISI distribution with linear time). Network parameters in this panel match those shown in Figure 2 above. **(B)** Increasing AAS input levels resulted in increased firing rate (left), higher dominant oscillation frequency (center) and a decrease in the number of spikes in the high and low modes of the ISI distribution (right) compared with the central mode, indicating that more spikes were becoming entrained to the dominant oscillation as AAS input increased. **(C)** Low but non-zero AAS input and no noise resulted in a very different dynamical state characterized by all neurons being entrained to a single low oscillation frequency (left). The LFP was characteristic of a rectangular wave (similar to a square wave but with a shortened or lengthened duty cycle) (center). Almost all ISIs were identical (right). **(D)** AAS input and all synaptic conductances in the network were set to zero and noise was added to induce random firing at 1 Hz (left). Mean firing rate was identical to that in **(A)** but the LFP was consistent with filtered noise, attenuated slightly at higher frequencies by the neural membranes (center). The ISI distribution was nominally exponential (inset, right) with the spike refractory period excluding short ISIs (main panel, right).

For the network with baseline AAS input level (equal to 1, as in Figures 2, 3A), LFP power was strongest at approximately 19 Hz, indicating the dominant oscillation frequency in this network (Figure 3A center). The ISI distribution appeared monotonic on a linear scale (Figure 3A right inset), but by exploiting the fact that very small ISIs are excluded by the refractory period, we plotted the ISIs on a log scale to give superior count resolution for short ISIs (Figure 3A right). On a log scale several peaks were apparent; the ISI distribution was clearly multi-modal. The central mode, occurring at just below 0.1 s (–1 on the log scale), was caused by the dominant 10–20 Hz oscillations. This mode could therefore be considered the “base mode” or default ISI of this network. In this respect, spikes occurring 0.05–0.1 s apart carried little information; these spike timings were predictable based on the observed dominant oscillation frequency, and in an information theoretic sense, the more predictable an event, the less information it conveys (Shannon et al., 1949). Unexpected, apparently random deviations from this base mode, however, can carry much larger amounts of information. Interestingly, for this network these deviations occurred in a manner suggestive of both integration and segregation of network elements, as follows:

The mode with shortest ISI, occurring at less than 0.01 s (–2.4 on the log scale), was caused when neurons integrated into assemblies. When a neuron received synaptic input from its local neighbors and from random long-range connections from other active assemblies elsewhere in the network, it would fire at much higher rates than baseline and become integrated by and into the network activity.

Similarly, the mode with longest ISI in the ISI distribution (Figure 3A, right), occurring at approximately 1 s (0 on the log scale) and beyond, was caused by neurons firing less often than expected given the network oscillation frequency, or equivalently, neurons being excluded from firing. The majority of the principal neurons in the network had lengthy periods of complete silence lasting 10 s or more despite the network as a whole displaying regular oscillations of much shorter periods.

### Input level from the ascending arousal system changed dynamical state

When constant input from the AAS was increased, several dynamical properties of the network were altered (see Figure 3B, with four times the AAS input). The network firing rate increased as a direct result of the increased input allowing a greater number of neuron assemblies to be simultaneously active. The dominant network oscillation frequency also increased, due to the stronger input causing faster depolarization of the neurons after each oscillation cycle; the oscillation peak shifted from 19 to 26 Hz (Figure 3B center). In the ISI distribution, more spikes occurred in the central baseline mode and fewer in the outlying short and long ISI modes (Figure 3B right). This change in distribution indicated greater predictability of the spike train—a greater proportion of spikes were being entrained to the overall dominant network rhythm and fewer were exhibiting enhanced integration with or segregation from this overall network activity. The reason for the reduced segregation is straightforward—with more neurons firing there are fewer opportunities for neurons to be silent for extended periods. On the other hand, the comparatively lower integration into assemblies is due to stronger inhibition (which itself is due to the increased overall activity in the network); each oscillation cycle is shorter, so fewer spikes are generated by each neuron in each cycle before the assembly is silenced by inhibition.

For some small but non-zero levels of input from the AAS, the network entered a very different state (Figure 3C, AAS input lowered to 0.75). The dominant oscillation frequency decreased markedly and virtually all neurons were entrained to the global oscillation. This state had a very low complexity, as can be seen from the single large peak in the ISI distribution at the dominant frequency. In this state, the spike times of all the neurons verged on wholly predictable. It is not immediately evident why this state arose. However, two observations may be relevant: (1) the state was not strongly stable, with dynamics settling into either low or high complexity regimes under initial conditions with very small differences, and (2) the state was abolished by noise (see Section Robustness to Noise, below). These observations suggest that the network dynamics were bistable in this region of phase space (a not-uncommon occurrence in non-linear dynamical systems).

To establish a control condition with Poisson firing, which reveals the differences in dynamics when network activity is dependent on extrinsic noise rather than on the network structure, AAS input and all synaptic conductances in the network were set to zero efficacy and sufficient noise was added to the neural membranes to induce random spiking activity at 1 Hz (Figure 3D, left). The LFP showed a power spectrum characteristic of low-pass filtered noise; higher frequencies were filtered by the membrane capacitance of each neuron (Figure 3D, center). The ISI distribution took the expected exponential shape curtailed by the spike refractory period. Poisson firing highlights the contrasts in dynamics between random activity and metastability, where the average firing rates were equal but the higher order statistics were dissimilar.

With the extrinsic noise removed and synaptic conductances reinstated (i.e., the network restored to standard baseline), AAS input was then varied from 0 to 10 in steps of 0.01, the mean firing rate for each instance was recorded, and the network *trapping time* was calculated (see Methods for details). Trapping time quantified the amount of time the network tended to remain in a given state before transitioning to a new state.

**Figure 4:**
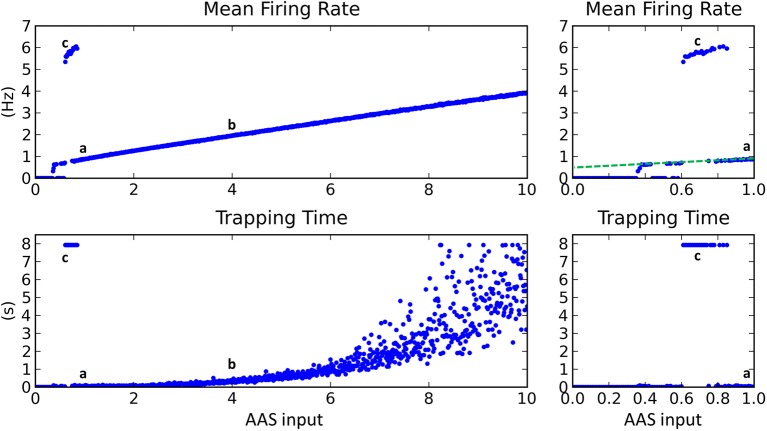
**Changing input level from the AAS dramatically affected network dynamics**. **Left panels**—AAS input levels from 0 to 10. **Right panels**—close-up on AAS input from 0 to 1. Cases a–c from Figure 3 are marked on the graph (top). Very low input levels below 0.6 usually resulted in no network activity, with sporadic instances of complex dynamics occurring for AAS input levels between 0.35 and 0.6 (see close-up panels on right). At input levels between 0.6 and 0.8, global entrainment at high firing rates but low oscillation frequencies emerged abruptly, again with some sporadic interspersed instances of complex dynamics. The sporadic large changes in firing rate and trapping time for low AAS input levels (between 0.35 and 0.8) are characteristic of network dynamics being bistable, with the random initial conditions for each network instance controlling which of the stable states the network settled into in each case. A small increment in AAS input could therefore result in a large change in dynamics, as can be seen in the close-up panels on the right. At AAS levels beyond 0.8, a sustained switch to complex dynamics occurred (i.e., the bistability vanished). At high levels of AAS input, firing rate and trapping time increased. Adding noise to the neural membranes removed the bistability and caused complex dynamics to be sustained for all levels of AAS input down to zero (dashed line, top right).

Mean firing rate increased linearly as AAS input was increased (Figure 4, top), except for low input values where firing rate was typically either zero or extremely high. The regions of zero network firing were generally for very low AAS input values near zero, while regions of very high firing rate occurred for slightly higher input levels between 0.6 and 0.8. The transformation between low and high firing rates was sudden, with no intermediate states. The dynamics in the high firing rate regions were as shown in Figure 3C—low complexity, global entrainment, and slow oscillations around 5 Hz. In these high firing rate regions the trapping time was high (Figure 4, bottom), signifying that the network state was not changing (marked as point **c** in Figure 4).

With increasing AAS input to just above 0.8, network dynamics again switched dramatically, in this instance to a state of low trapping time (high complexity) and low firing rate, as shown in Figure 3A (marked as point **a** in Figure 4). Trapping time and firing rate increased with further rises in AAS input (point **b** and beyond). The firing rate increase has been explained above. The trapping time increase is related to, and perhaps caused by, the increase in firing rate; specifically, by the increase in the number of simultaneously-active assemblies. When a greater number of assemblies are active at any given time, there are fewer long-range connections that project to currently-inactive assemblies. Only inactive assemblies have a chance of switching to an active state and changing the overall state of the network. The consequence is that, as firing rate increases, there is decreasing probability of a change in state at any given time, as reflected in the increasing trapping time.

### Connectivity impacts dynamics

**Figure 5:**
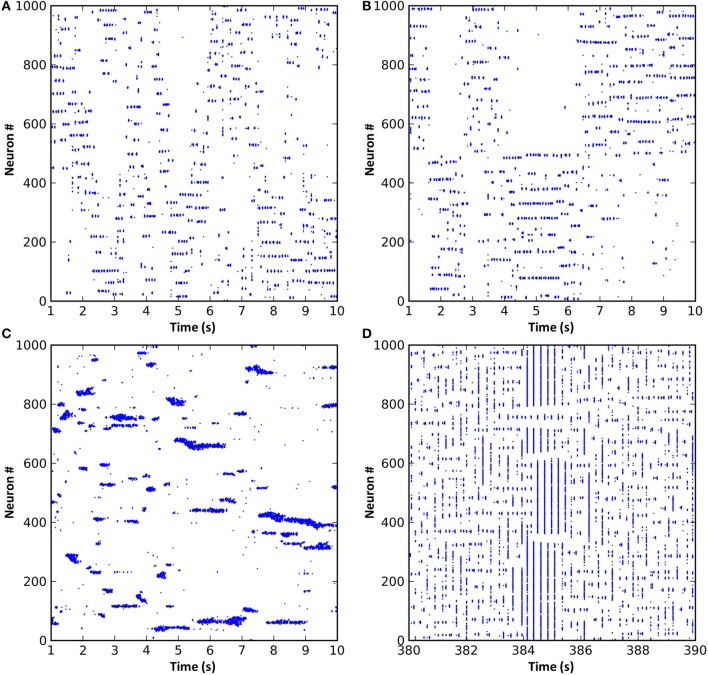
**Modifications to the synaptic connection schemes resulted in changes to the network dynamics**. **(A)** Long range connection probability based entirely on distance between neurons resulted in propagating waves of activity. **(B)** Dividing the neurons into two groups where neurons within a group were more likely to be connected resulted in a dynamic switching of activity between groups. **(C)** Reducing the radius of local inhibition to less than that of local excitation resulted in the formation of variably-sized neuronal assemblies and much faster oscillations. **(D)** If short-term synaptic dynamics are modeled and excitation and inhibition strength increased, a network that exhibits spontaneous entry to and exit from seizure states can result.

In all of the above simulations, long range cortical connections were set randomly with uniform probability. It is also possible to base connection probability on distance between the cells, so that cells that are nearer have a greater chance of having a long range connection between them. Such a connection scheme resulted in “waves” of activity propagating through the cortical cells (Figure 5A). These waves arose due to the connections from any active region in the network projecting most densely to regions that were immediately adjacent. These adjacent regions, receiving the strongest input, were the most likely to become active next. As this process repeated, the result was a wave of activity propagating through the network. However, since the propagation was chaotic, the speed, number and even the direction of the propagating waves could vary unexpectedly. Alternatively, by dividing cortical neurons into two groups such that intergroup long range connections were less likely than intragroup, activity would unpredictably switch between the groups (Figure 5B). The activity switch occurred for a similar reason to the activity waves in Figure 5A, except that the connection probability within each group was fixed and equal between all neurons, causing all neurons within a group to have equal chance to become active.

Reducing the radius of local inhibition resulted in variable neuronal assembly sizes and much faster oscillations (Figure 5C). Assembly sizes varied because the reduced radius of inhibition was unable to contain the surrounding excitation, meaning that adjacent assemblies could link together and form larger super-assemblies. Oscillation frequency increased because local inhibition could no longer provide a stabilizing effect on the network dynamics; instead, local excitatory connections could form tight recurrent loops limited in frequency only by axonal conduction delays and membrane dynamics. Finally, increasing local inhibition and excitation strength and adding short-term plasticity (STP) to the excitatory synapses resulted in network dynamics which would spontaneously and intrinsically enter and exit seizure states (Figure 5D). STP seems to be involved in the generation of seizure-like dynamics in the model, since networks without STP do not exhibit these seizure states. Seizure onset was caused by chance synchronous firing, during one oscillation cycle, of a larger number of neurons than normal, simultaneously facilitating a large number of synapses, which pushed the network into a hyper-synchronous state that was then perpetuated (often beyond the facilitation time constant) by feedback through the thalamic matrix connections. Seizure termination also seemed to occur by chance, with a spike from a cell either not involved in the seizure or at a time not synchronized with the seizure oscillations. Such a “wayward” spike could sufficiently affect the timing of subsequent spikes in the network to break the hyper-synchronicity. However, a deeper understanding of the mechanism or mechanisms involved in seizure onset and termination would require further studies that are beyond the scope of the current paper.

In the version of the CXC network presented in this paper, the pedunculopontine, reticular and intralaminar nuclei are each modeled as unitary entities with no smaller components or sub-nuclei. While this simplification is adequate to demonstrate the functional involvement of these regions in long range segregation of cortical activity and facilitation of complex dynamics, in reality these nuclei are comprised of distinct components with distinct connection patterns. In particular, the “non-specific” matrix projections from the IL to the cortex may in fact have specific synaptic targets (Groenewegen and Berendse, 1994). This specificity has consequences for cortical dynamics: rather than cortical regions competing uniformly for activity, the competition (segregation) occurs in interconnected, hierarchically-arranged overlapping pockets of varying sizes across the entire cortex. With such a connection paradigm, total cortical activity remains controlled at all times, but inter-regional competition can vary in complex ways based on IL connection patterns and precise patterns of cortical activity at any given time. The result is that the repertoire of cortical dynamics is potentially even richer than presented here.

The network is not limited to linear (1-dimensional) layouts; 2-dimensional, and greater, network arrangements work equally as well (see online^2^ for details). Overall, this and the above preliminary results (Figure 5) suggest a strong impact of network connectivity on the ensuing neural dynamics that warrants further investigation. Understanding how neural dynamics are influenced by specific connection structures in the brain such as the directed connections between regions at the macro scale, intra-regional connectivity inside structures like the hippocampus at the meso scale, and detailed neural circuitry at the micro scale, is critical to understanding how these structures perform their diverse functions.

### Robustness to noise

To test the robustness of the network dynamics to noise, sufficient membrane noise was added to all principal cells to induce random spiking at 0.1 Hz in each cell; that is, spiking occurred at 100 Hz across the network of 1000 cells. (Note that this is a very different experiment to that depicted in Figure 3D, where the network was effectively disabled by setting synaptic efficacies to zero prior to the injection of noise.) In the case of adding noise to the functioning network, there was no significant change in the mean firing rate, integration and segregation, or the trapping time for most AAS input levels. However, for AAS input between 0 and 0.8, the transitions to low complexity states with high mean firing rates and high trapping times did not occur; instead, complex dynamics continued for all input levels down to zero (Figure 4, dashed line in top right panel). This continuation of complex dynamics was due to random spiking activity adding to the total activity in the network, similar to sustained higher AAS input, and holding the network in a metastable dynamical state. Crucially, the network's ability to maintain metastability and long range segregation of activity was not compromised by noise.

## Discussion

### Segregation, metastability, and the thalamocortical matrix

The primary finding in the current study has been that the AAS-IL-RN circuit may have the ability to globally segregate cortical activity and maintain metastable cortical dynamics, with no need for injected noise or input perturbations, and that the global cortical state could be strongly modulated by brainstem input to the thalamus. In the absence of long-range inhibition, and the absence of long-range excitation onto local inhibitory interneurons, the long-range competition and network segregation observed in the CXC could only occur through the lowering of excitation from the IL. Local and long-range corticocortical connections in the CXC network were not strong enough to sustain activity at low firing rates—a depolarizing input from the IL was required to boost neural membrane potentials sufficiently close to threshold so that a small number of presynaptic spikes was capable of causing a spike in the postsynaptic neuron. If total activity in the CXC network increased, tonic depolarizing input from the IL decreased, and neurons or assemblies that would have fired due to convergent input from other active assemblies would then be unable to fire. This is competition through *effective* inhibition (inhibition that occurs via the withdrawal of tonic excitation), controlled by the total amount of activity across the network. Simultaneous, transient, unpredictable, recurring integration and segregation of activity in the CXC network resulted in the observed on-going metastability. Without these thalamocortical matrix connections, sustained metastability at low firing rates, long range competition, and global segregation of cortical activity would not be possible.

The activity states observed in the CXC network have analogs in real brains. The slow oscillations and global coupling seen at low AAS input levels in the CXC network are similar to the strong delta oscillations and very low dynamical complexity observed during deep (non-REM) sleep (Massimini et al., 2005; Murphy et al., 2009). In mammals, AAS input levels, particularly input from the pedunculopontine nucleus (PPN, or sometimes PPT or PPTg) are significantly reduced during periods of non-REM sleep (Moruzzi and Magoun, 1949; Reese et al., 1995; Jones, 2003), with slow delta oscillations at 0.5–4 Hz the predominant neural activity signature of this state. The transition between sleep and active cortical states is thought to be driven by cholinergic neuromodulation. However, there is likely to be more than one mechanism involved, and this result in the CXC network suggests that the transition could be assisted by the lowering of AAS input; supporting the switch from a state of high to low dynamical complexity.

At slightly higher AAS input levels than those required to generate sleep-like dynamics, the CXC network switches into a low firing rate, high complexity state. This state is analogous to awake states of low cortical arousal and metastability seen in real brains at intermediate levels of PPN activity, and observed during quiet relaxation (Stam et al., 1999). The characteristic neural activity signature of this state is relatively slow alpha oscillations (8–12 Hz, just lower than the dominant oscillation frequency observed in the CXC network) seen over large parts of the cortex. Increasing AAS input beyond this level in the CXC network resulted in increased firing rates, increased dominant oscillation frequency and increased trapping time—the length of time for which neural assemblies were stable—while decreasing the dynamical complexity. The decrease in complexity arose due to more spikes being entrained to the dominant oscillation and fewer spikes exhibiting either integration into assemblies or segregation from network activity. These states in the CXC network may correspond to states of attention, arousal and vigilance in real brains when AAS input is known to be maximal.

### Default mode network

Free association and daydreaming tend to occur when the brain is not actively processing sensory stimuli or task-related events, and as such these states are correlated with activity in the default mode network (Mason et al., 2007; Buckner et al., 2008; Christoff et al., 2009). It follows that when the brain switches state from free association to attention, activity tends to switch from the DMN to other parts of the cortex. The regions of the cortex involved in the DMN are more strongly connected to each other than they are to the rest of the cortex (Buckner et al., 2008). We have shown that when a similar connection strategy is employed in the CXC network—dividing the cortical neurons into groups where intergroup connections are less likely than intragroup—activity dynamically and unpredictably switches between groups.

In the CXC network, activity tends to concentrate in one group at a time because the recurrent long range connections required to sustain ongoing activity (Stratton and Wiles, 2010b) are focused within groups. Switching between groups is driven by a combination of the long range connections and the input from the AAS; when several long range inputs to a new group are activated simultaneously, perhaps coinciding with a decrease in activity of the currently-active group due to either habituation or random activity fluctuation, then input from the AAS can be sufficient to ignite a larger number of assemblies in the new group. Activity in the new group then competes for persistence with the current group through the segregation process mediated by the AAS and the thalamic matrix connections, and if activity in the new group is strong enough then it will dominate and an activity switch occurs. This dynamic switching in the CXC network provides a parsimonious explanation and inherent mechanism for activity in the DMN and other cortical regions being mutually exclusive despite the limited connectivity (especially inhibitory connectivity) between them. In the brain, the timing of switches in activity between the default mode and task-positive networks is likely also influenced by task requirements and external events in the perceived environment, rather than being purely chaotic, but the segregation principle remains the same.

### Consequences for brain function

Sustained firing of neural assemblies has been proposed as the neural substrate of working memory in the cortex (Wang, 1999; Pesaran et al., 2002; Jensen, 2006; Jensen et al., 2007). In the CXC network, the state of higher firing rates, increased trapping times and faster oscillations is analogous to a state of increased vigilance, attention and working memory in the cortex (Oken et al., 2006), driven by an increase of input from the AAS (specifically the PPN). There is ample evidence that increasing input from the AAS causes increasing cortical activation in general (Jones, 2003), and that firing rate and oscillation frequency also increase specifically for those neurons representing attended stimuli (Fries et al., 2001). There is also some evidence that increased vigilance does indeed reduce spiking variability (meaning that complexity of spiking patterns is also reduced) (Falkner et al., 2013). The converse state of low firing rates, short trapping times and high complexity in the CXC network is driven by lower AAS input levels and is associated with slower oscillations. This state is analogous to fluid states of mentation such as mind-wandering or daydreaming (Laufs et al., 2003; Mason et al., 2007; Buckner et al., 2008; Christoff et al., 2009) where working memory is not heavily utilized and mental associations arise freely and apparently randomly due to the increased variability of the neural dynamics.

The CXC network has a clear dichotomy between relaxed free association and vigilant attention; these modes cannot occur together because they are distinct dynamical states of the thalamocortical system, driven by changing input levels from the AAS. Dynamical states of low complexity are necessary for stable maintenance of working memory, with the trade-off that fewer potential states will be visited due to longer network trapping times. Dynamical states of high complexity and short trapping times are necessary for exploring more of the possible state space combinations of neural representations, supporting mind-wandering and free association, but are ineffective when focussed attention and working memory are required. Based on the model's behavior, we conjecture that states of high and low complexity are both useful but cannot co-occur, so the brain switches between them as need and opportunity arises.

Several predictions about neural dynamics can be made from the model:

1. Overall cortical activity should increase approximately linearly with increasing stimulation from the AAS. This correlation could possibly be measured experimentally using fMRI.
2. Increasing AAS input should cause increased trapping time in the cortex (i.e., longer activation of neural assemblies and slower transitions between cortical states). This relationship could be quantified using fMRI or multi-electrode electrophysiology recordings.
3. Increasing AAS input should also reduce the complexity (increase the predictability) of dynamic cortical activity patterns. Predictability can be quantified by calculating the entropy of or the mutual information in recorded spike trains (Dorval, 2008).

What can the CXC network tell us about computation in the brain, rather than simply brain dynamics? To adequately address this question requires consideration of both representation (how does brain activity represent information?) and learning (how does this information come to be in the brain?); these are substantive and intricately connected topics which are the focus of much research today. In its current form, the CXC network offers mechanisms behind the intrinsic, autonomous generation of metastability that seem to be required for complex thought (Binder et al., 1999; Mazoyer et al., 2001; Breakspear et al., 2003; Buzsáki and Draguhn, 2004; Fries, 2005; Tognoli and Kelso, 2014) and for the stable maintenance of dynamic neural assemblies required for working memory (Wang, 1999; Pesaran et al., 2002; Jensen et al., 2007). These apparently conflicting requirements are addressed through the control exerted over network dynamics by the level of input from the AAS to the thalamus. With these mechanisms in place, the dynamics observed in the CXC can potentially inform existing and perhaps even all-new theories of information transmission and transformation in the brain. Likewise, theories of learning, representation and mental processing can be tested in, and may lead to refinements of, the CXC. Initial questions include:

- Can repeated activity sequences be embedded in the network and spontaneously replayed—spontaneous sequence replay has been seen in animals during sleep and decision-making.
- Can plasticity mechanisms bias network dynamics to functionally integrate given cortical regions on demand (based on specific internal or external triggers)—this would help answer how the brain learns to functionally connect brain regions for the propagation of information as required.

Subsequent questions may address more complicated issues, such as how complex representations can be constructed in dynamical neural assemblies and utilized by the brain for purposeful computation. The ultimate goal of this future research will be to link the observed network dynamics with computational states in the brain. While realization of this goal is clearly distant, studying the CXC network will potentially lead in this direction.

### Pathological dynamics

Many brain disorders are associated with alterations to cortical dynamics, the DMN and the AAS (Garcia-Rill, 1997; Schnitzler and Gross, 2005; Uhlhaas and Singer, 2006; Buckner et al., 2008; Fröhlich et al., 2008; Fox and Greicius, 2010; Zhang and Raichle, 2010). We have demonstrated the dependence of autonomously-generated metastable dynamics on network connectivity in these regions. Because up to 99% of cortical connections derive from the cortex (Braitenberg and Schüz, 1998) and only a small fraction of the brain's energy consumption is used for the processing of external events (Raichle and Mintun, 2006; Zhang and Raichle, 2010), by far the majority of cortical activity originates in and is driven by other cortical activity. Changes in cortical structure driven by either learning or disease may therefore affect ongoing dynamics in unpredictable ways. Furthermore, these changes in dynamics may often lead to further structural change, creating a recursive dependency between dynamics and structure that could possibly lead to pathological states, such as in epilepsy, sleep disturbance, schizophrenia and many other brain disorders. By modeling autonomous metastable activity in the AAS-IL-RN circuit, this recursive chain of structural-dynamical co-dependence remains intact, allowing the investigation of how this dependence may lead to pathological states. Such studies are directions for future research with the CXC model.

### Advances in the CXC model

The thalamocortical matrix loop modeled in the CXC network is reminiscent of a winner-take-all (WTA) mechanism, however it is more accurately described as implementing winnerless competition (WLC). In WLC, a clear winning neuron or neural assembly never emerges from the competition; instead, each winner is immediately displaced by the next, resulting in continuously-evolving complex dynamic activity patterns (Akrami et al., 2012; Rabinovich et al., 2012). WLC can occur in networks of neurons mutually connected with inhibitory synapses, as observed in some simple animals during the generation of unpredictable behavior (Levi et al., 2005). It has also been shown analytically to occur in networks of neurons connected with slow global inhibition (Ermentrout, 1992). One of the insights offered by the CXC model is how the thalamocortical matrix can provide the necessary global inhibition to implement WLC for the control of complex dynamics across the cortex.

Random networks with balanced local excitation and inhibition have previously been shown to exhibit chaotic dynamics (Van Vreeswijk and Sompolinsky, 1996). Since then, studies have shown that networks connected using small world principles can also exhibit complex dynamics (Sporns et al., 2000b; Sporns and Tononi, 2001; Roxin et al., 2004; Riecke et al., 2007; Shanahan, 2008). However, for common topologies of these networks, the regions of parameter space where metastability was evident was small (Breakspear et al., 2003; Shanahan, 2008). More recently it has been shown that activity-dependent synaptic depression (a short-term decrease of synaptic efficacy based on postsynaptic activity) can massively enlarge the critical region where metastability occurs (Levina et al., 2007), and that the voltage-dependence of synaptic currents can stabilize complex dynamics for long periods of time (Kumar et al., 2008). Most recently, networks organized into hierarchical modules, where intra-module connections are abundant and inter-module connections are sparse, have been studied (Rubinov et al., 2011; Wang et al., 2011). Unlike previous networks, these latest networks (Kumar et al., 2008; Rubinov et al., 2011; Wang et al., 2011) can exhibit *irregular* sustained activity at low firing rates. For all of the above-mentioned networks, however, some or all of these questions remain open:

- How can complex dynamics be sustained indefinitely?
- How can low average firing rates, as seen in cortex, be obtained?
- Can dynamics be maintained without injection of extrinsic noise?
- Can very long interspike intervals (tens of seconds) be achieved?
- How can explicit segregation of activity (as against just a lack of integration) be accomplished?

In the current study, we have shown how the thalamocortical matrix connections control cortical dynamics and resolve the above issues. Complex metastable dynamics in the CXC network continue indefinitely with no need for injected noise, at low firing rates and with very long ISIs occurring frequently. Most importantly, the matrix connections can cause explicit dynamic segregation of network activity through withdrawal of tonic excitation—a process we have termed *effective* inhibition. Effective inhibition leads to activity between weakly-connected regions (such as the default mode and task positive networks) being significantly anticorrelated rather than simply uncorrelated. A mechanism by which effective inhibition can arise globally across the cortex has not previously been suggested.

## Conclusion

The first and main result of this paper—that the global inhibition required for winner-take-all dynamics can be implemented by the diffuse thalamocortical loop—is not immediately obvious from neurophysiological observation, since there is little large-scale long-range inhibition within the loop. For this reason we have termed the process ‘effective inhibition’. Secondly, we show how changing tonic input levels from the ascending arousal system to the thalamus can change the dynamical state in the cortex (Section Input Level from the Reticular Activating System Changed Dynamical State). This result explains previous observations concerning how AAS input affects cortical activity (such as state changes between sleep, wakefulness and vigilance) and makes several novel predictions (Section Consequences for Brain Function). Thirdly, we show how cortical connectivity affects the sustained dynamics (Section Connectivity Impacts Dynamics). These sustained activity patterns can potentially be understood in terms of WTA dynamics, and we have demonstrated that the ‘effective inhibition’ paradigm, with its fundamentally different mechanism, is capable of supporting these patterns. When the network was structured into task-positive and DMN cortical regions with dense local and sparse long-range connections, we additionally showed how activity could intrinsically alternate between the groups based on chaotic dynamics with no extrinsic noise. We argue that such intrinsic alternation provides a plausible explanation for the dynamical segregation of the DMN from other cortical regions. We have shown that the network is robust to noise, but importantly does not require noise for the generation and maintenance of a complex, ongoing brain state.

Previous studies have examined networks that exhibited complex dynamics, but these networks were unable to achieve long range segregation of activity. In contrast, the CXC network achieves both segregation of activity and metastability through the global control of the RN acting through IL. Without global control, network activity reduces to numerous interconnected pockets of activity that can mutually integrate due to activity propagating through the long range connections, but cannot mutually segregate.

### Conflict of interest statement

The authors declare that the research was conducted in the absence of any commercial or financial relationships that could be construed as a potential conflict of interest.

## Footnotes

^1^While the subcortical structure of the CXC is more complicated than that presented in this earlier paper, it fulfills the same function and the extra detail has been included for biological fidelity. Parameter sensitivity is therefore not significantly altered in the CXC.

^2^For an animated example of 2-dimensional cortical dynamics in the CXC see https://dl.dropboxusercontent.com/u/70159706/gtrajectory_out.avi.
